# Pasteurized *Akkermansia muciniphila* increases whole-body energy expenditure and fecal energy excretion in diet-induced obese mice

**DOI:** 10.1080/19490976.2020.1737307

**Published:** 2020-03-13

**Authors:** Clara Depommier, Matthias Van Hul, Amandine Everard, Nathalie M. Delzenne, Willem M. De Vos, Patrice D. Cani

**Affiliations:** a Metabolism and Nutrition Research Group, Louvain Drug Research Institute, Walloon Excellence in Life Sciences and BIOtechnology (WELBIO), UCLouvain, Université Catholique De Louvain, Brussels, Belgium; b Laboratory of Microbiology, Wageningen University, Wageningen, The Netherlands; c Human Microbiome Research Program, Faculty of Medicine, University of Helsinki, Helsinki, Finland

**Keywords:** *Akkermansia muciniphila*, pasteurization, mice, obesity, indirect calorimetry, brown adipose tissue metabolism, white adipose tissue metabolism, perilipins, motor activity, intestinal turnover, carbohydrates absorption

## Abstract

Accumulating evidence points to *Akkermansia muciniphila* as a novel candidate to prevent or treat obesity-related metabolic disorders. We recently observed, in mice and in humans, that pasteurization of *A. muciniphila* increases its beneficial effects on metabolism. However, it is currently unknown if the observed beneficial effects on body weight and fat mass gain are due to specific changes in energy expenditure. Therefore, we investigated the effects of pasteurized *A. muciniphila* on whole-body energy metabolism during high-fat diet feeding by using metabolic chambers. We confirmed that daily oral administration of pasteurized *A. muciniphila* alleviated diet-induced obesity and decreased food energy efficiency. We found that this effect was associated with an increase in energy expenditure and spontaneous physical activity. Strikingly, we discovered that energy expenditure was enhanced independently from changes in markers of thermogenesis or beiging of the white adipose tissue. However, we found in brown and white adipose tissues that perilipin2, a factor associated with lipid droplet and known to be altered in obesity, was decreased in expression by pasteurized *A. muciniphila*. Finally, we observed that treatment with pasteurized *A. muciniphila* increased energy excretion in the feces. Interestingly, we demonstrated that this effect was not due to the modulation of intestinal lipid absorption or chylomicron synthesis but likely involved a reduction of carbohydrates absorption and enhanced intestinal epithelial turnover.

In conclusion, this study further dissects the mechanisms by which pasteurized *A. muciniphila* reduces body weight and fat mass gain. These data also further support the impact of targeting the gut microbiota by using specific bacteria to control whole-body energy metabolism.

## Introduction

Obesity and its ensuing metabolic disorders, including insulin resistance and cardiometabolic complications, represent a growing epidemic and economic burdens for public health authorities.^1,2^ Moreover, it is currently largely accepted that the gut microbiota can influence whole-body metabolism by affecting energy balance.^3^ The recognition that the gut microbiota is a key factor involved in the development of obesity-related disorders raises the prospect to study single intestinal microorganisms and combinations thereof with potential benefit for health.^4-6^ In this context, several studies have highlighted the multiple health-promoting effects of *Akkermansia muciniphila*, an intestinal mucin-degrading symbiont that is considered currently as a next-generation beneficial microbe.^7^ *A. muciniphila* belongs to the phylum Verrucomicrobia, is a Gram-negative bacteria, and represents 1-5% of the total human microbiome in healthy conditions.^8^ The abundance of *A. muciniphila* is severely reduced in several pathological conditions (i.e., obesity, type 2 diabetes, inflammatory bowel diseases, and appendicitis).^9-15^ We demonstrated that oral administration of *A. muciniphila* counteracts diet-induced obesity (DIO) in mice.^16^ Importantly, the beneficial effects of the bacteria on cardiometabolic features were thereafter supported by others using various pathological murine models.^17-22^ Recently, we discovered that pasteurization of the bacteria (i.e., treatment for 30 min at 70°C) made it more effective than the live bacteria in preventing DIO in mice.^23^ This finding was recently confirmed in a first proof-of-concept pilot human intervention.^24^ In addition to the major observation that treatment was well tolerated and declared safe in individuals suffering from metabolic syndrome, we reported that pasteurized *A. muciniphila* significantly alleviated the worsening of the disease observed in the placebo condition.^24^

Several studies have reported positive correlations between the abundance of *A. muciniphila* and energy expenditure or thermogenesis.^25-27^ However, none of these studies investigated its impact on whole-body energy metabolism by using indirect calorimetry, and the effect of pasteurized *A. muciniphila* on whole-body energy features remains unknown. Therefore, we first aimed to confirm the protective effect of pasteurized *A. muciniphila* in a DIO model. Second, we aimed to determine whether this protective effect was mediated through the modulation of energy expenditure and physical activity by using the gold standard method (i.e., indirect calorimetry). Mice were housed in metabolic chambers during the last seven days of a 5-week experiment consisting of daily oral gavage of pasteurized *A. muciniphila* concomitant to a high-fat diet (HFD) exposure.

## Results

### Pasteurized A. muciniphila prevents diet-induced obesity

**Figure 1.**
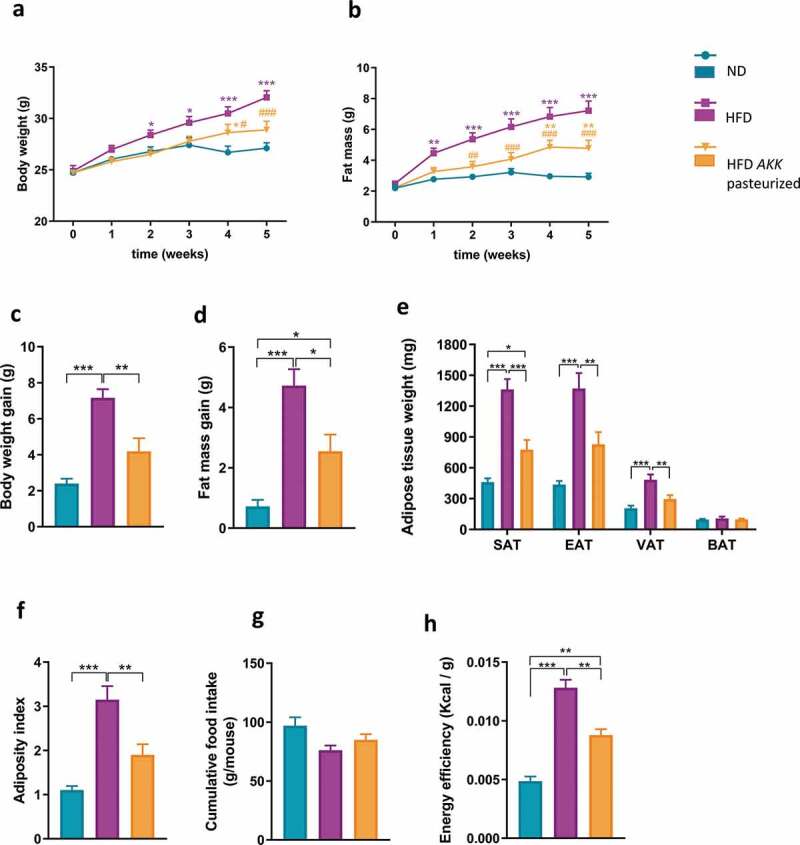
Pasteurized *A. muciniphila* modulates body composition and reduces energy efficiency in HFD-fed mice.

Pasteurized *A. muciniphila* significantly reduced HFD-induced body weight gain and fat mass gain (Figure 1a–d), without affecting cumulative food intake (Figure 1g). More specifically, the fat mass gain was already significantly lower as early as 2 weeks after the beginning of the treatment with pasteurized *A. muciniphila* (Figure 1b). Consistently, at the end of the experiment, we found that the different adipose depots (SAT: subcutaneous; EAT: epididymal; VAT: visceral) were significantly smaller in the treated group, except for the brown adipose tissue (BAT), which remained similar between the three groups (Figure 1e). Hence, the total adiposity index was significantly lower in the treated mice (Figure 1f). Given that the animals were gaining less weight without changes in the total energy ingested, we observed a significant reduction in energy efficiency in the treated group compared to the HFD group (Figure 1h). Of note, the absolute lean mass did not vary significantly between groups over the course of the experiment (Sup Figure 1a). However, in concordance with the evolution of the fat mass in the different group, when expressing the lean mass as the percentage of body weight, this parameter significantly decreased over time in the HFD group when compared to both control group and treated group (Sup Figure 1b).

### Pasteurized A. muciniphila enhances energy expenditure, oxygen consumption and physical activity in HFD mice

**Figure 2.**
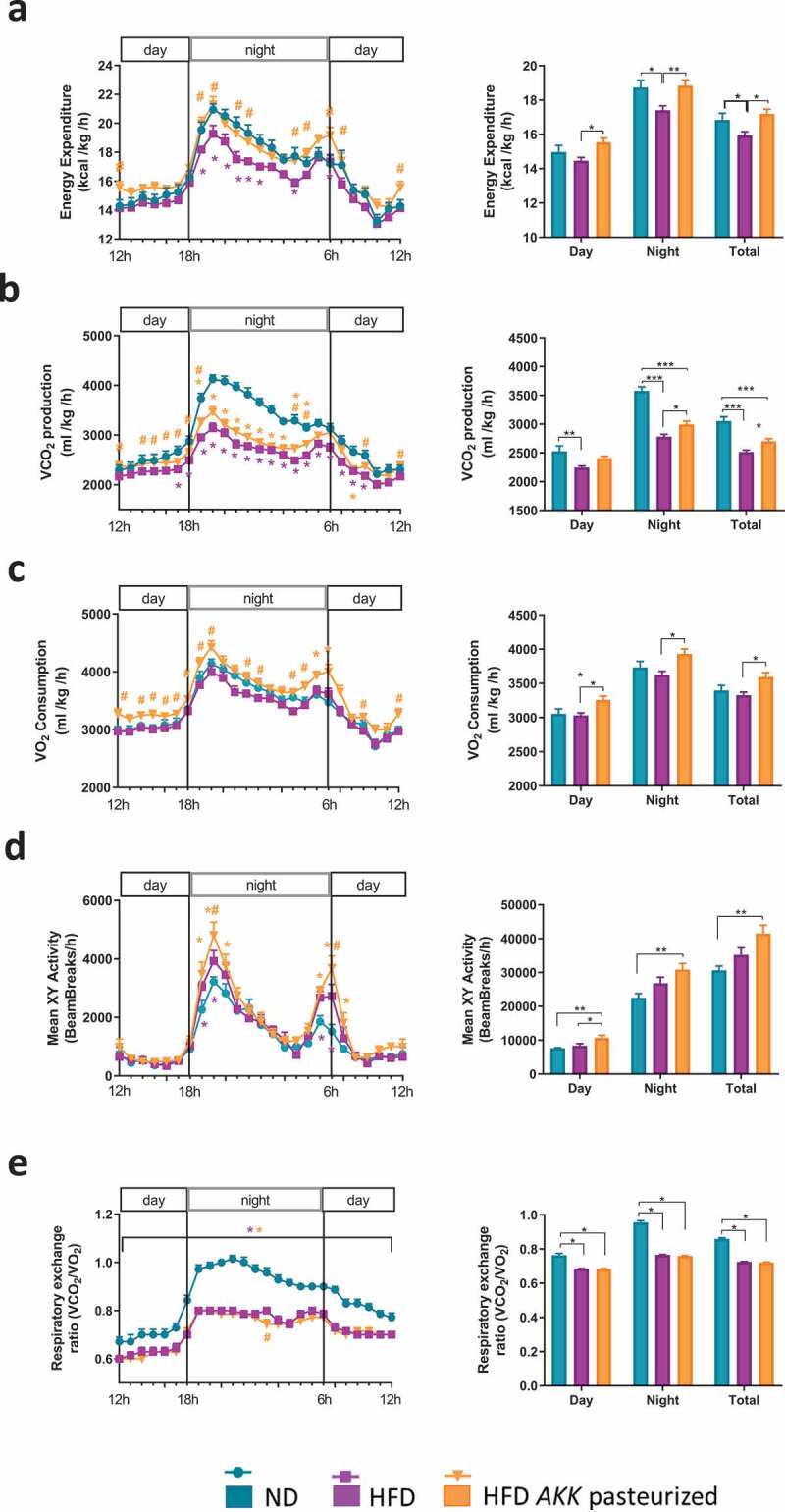
Pasteurized *A. muciniphila* increases energy expenditure and physical activity in HFD-fed mice.

To further dissect the mechanism explaining why mice treated with pasteurized *A. muciniphila* are gaining less weight and fat mass, mice were housed individually in metabolic chambers during the last week of experiment. The results from indirect calorimetry revealed that HFD-fed mice exhibited a reduced energy expenditure compared to their lean littermates (Figure 2a). The effect of the HFD was completely abolished by the treatment with pasteurized *A. muciniphila*, since mice were spending the same amount of energy than the normal diet (ND)-fed mice (Figure 2a). This protection was mirrored by a trend in increasing CO_2_ production (Figure 2b) and a significant enhancement of VO_2_ consumption in treated mice (Figure 2c). In addition, mice treated with pasteurized *A. muciniphila* exhibited a significant increase in locomotor activity when compared with control, while this parameter was not altered by the exposition to HFD (Figure 2d). As expected, the respiratory exchange ratio (RER) was significantly lower in HFD-fed groups (Figure 2e). This outcome was in agreement with a preference for lipid utilization in HFD-fed groups and glucose utilization in ND-fed mice.

### Pasteurized A. muciniphila does not modulate SAT beiging or BAT function

**Figure 3.**
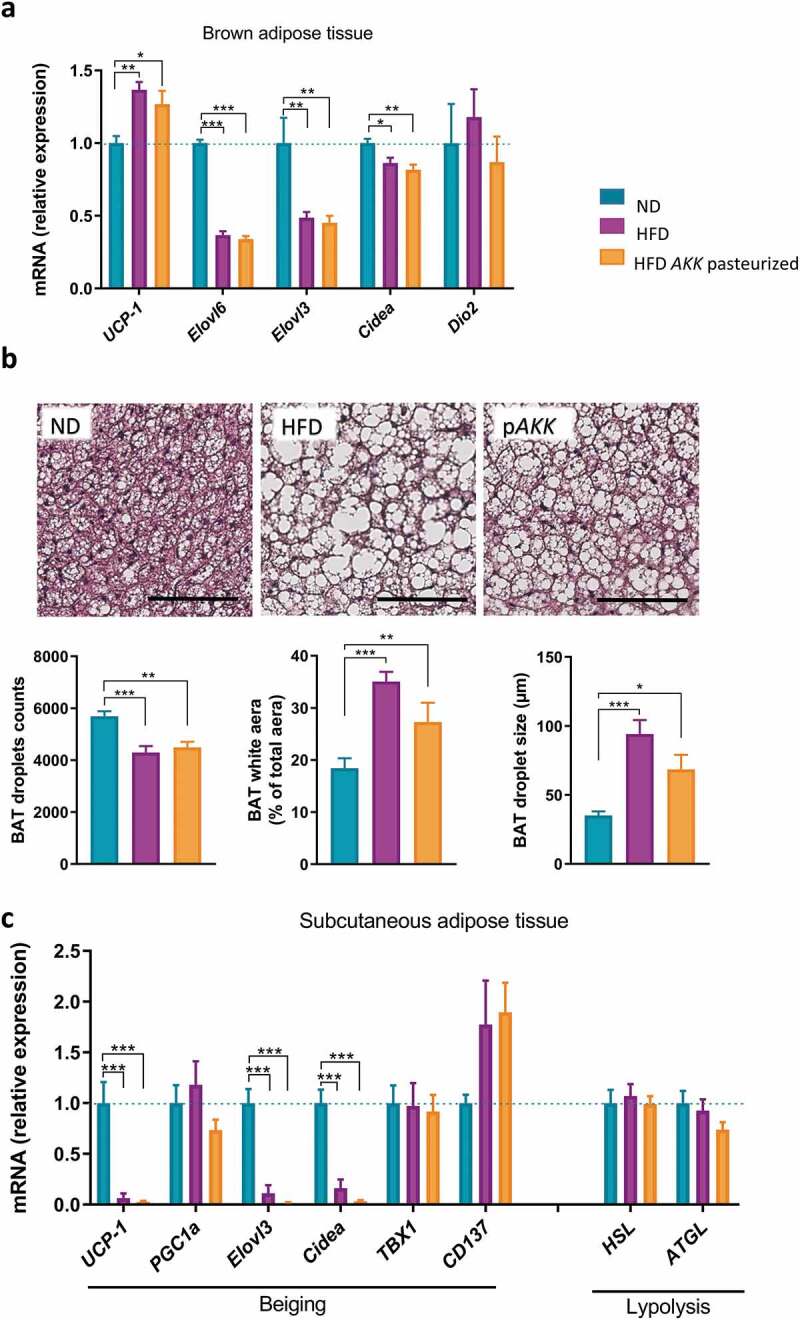
Pasteurized *A. muciniphila* does not modulate markers of beiging in the SAT and metabolism of BAT in HFD-fed mice.

To determine the contribution of thermogenesis in increased energy expenditure upon pasteurized *A. muciniphila* supplementation, we quantified the mRNA expression of several markers related to BAT function. As depicted in Figure 3a, an HFD induced a strong and significant downregulation of Elovl6, Elovl3 and, Cidea, while UCP-1 expression was enhanced and Dio2 expression was unchanged. We did not find a marked treatment effect on any of those markers. Histological analysis of BAT further validated those data; we noticed an enhanced whitening process upon HFD, as defined by development of enlarged lipid droplets. Pasteurized *A. muciniphila* treatment decreases the whitening and the lipid droplet size of the BAT by approximately 30–40% but this effect did not reach significance (*P* = .11 versus ND) (Figure 3b). To further extend our initial hypothesis, we investigated markers of beiging program and lipolysis in the SAT. We observed an important downregulation of the expression of UCP-1, Elovl3, and Cidea upon an HFD, while TBX1 and PGC1α expression levels were unaffected either by the HFD or treatment. The expression of CD137 was somewhat higher in the HFD-fed groups, but this did not reach statistical significance (*P* = .2 versus ND). Treatment with pasteurized *A. muciniphila* did not significantly modulate those alterations. Finally, both markers of lipolysis (HSL and ATGL) were statistically unaffected either by diet or treatment (Figure 3c).

### Pasteurized A. muciniphila influences expression of lipid-droplet regulator associated proteins

**Figure 4.**
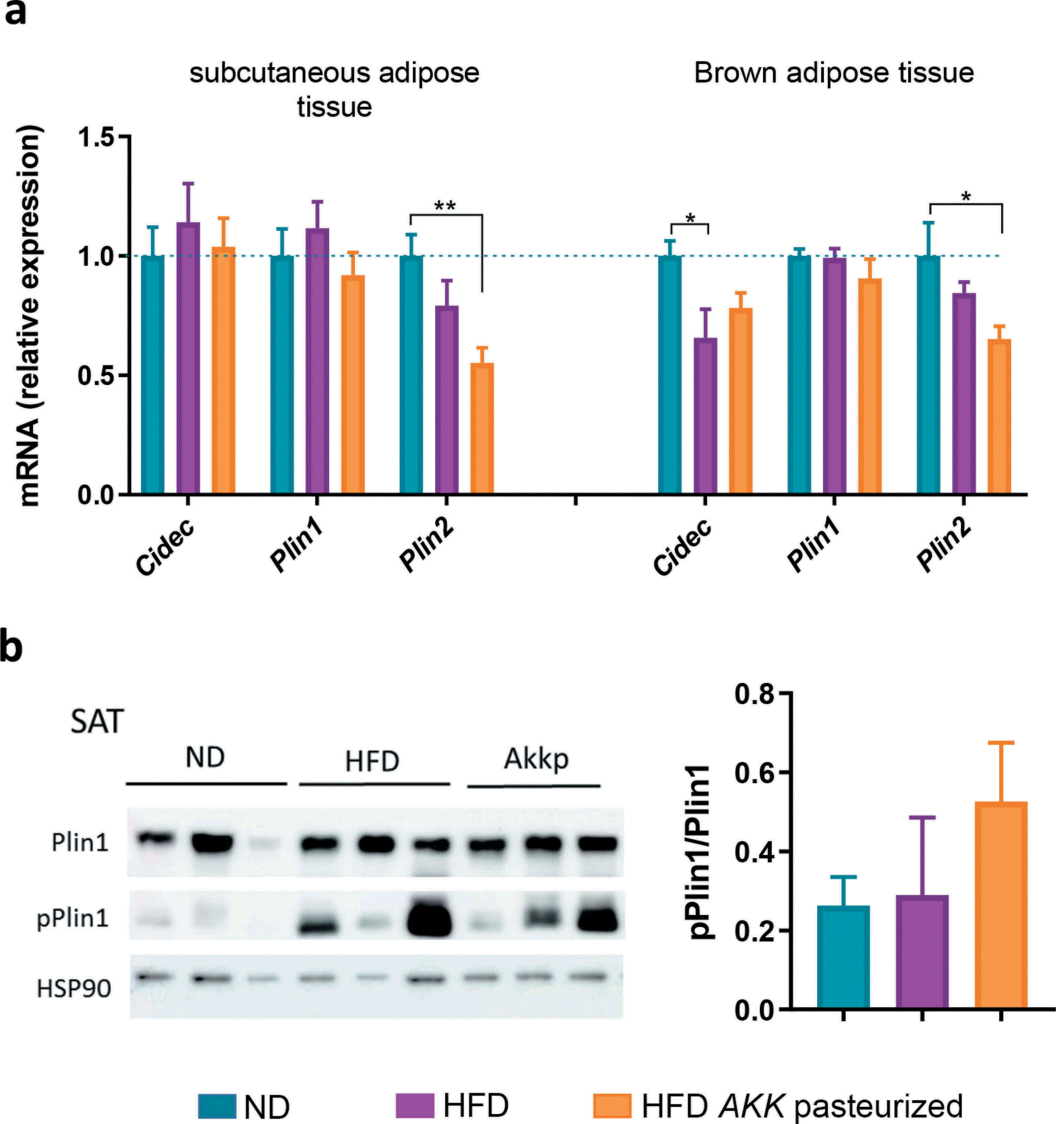
Mice treated with pasteurized *A. muciniphila* are characterized by a specific reduced expression of perilipin2 in adipose tissue.

Lipid droplet surfaces are decorated by the presence of specific proteins that play important roles in the regulation of adipocyte metabolism, notably with regards to caloric input, storage, morphologic features, and energy demands.^28^ Consistent with this notion, we investigated whether pasteurized *A. muciniphila* might influence those lipid metabolism regulators in SAT and BAT. Perilipin1 expression was found to be similar between groups in both tissues. Since phosphorylation is known to be a strong inducer of perilipin1 cellular action, we also quantified phosphorylated-perilipin1 protein levels in SAT and found no major diet or treatment effect (Figure 4a,b). Whereas perilipin1 was found to be unaffected, a significant downregulation of perilipin2 was observed upon treatment in both tissues when compared to control (Figure 4a). As perilipin2 is ubiquitous, we also quantified its expression in muscles, as well as markers of oxidative metabolism. By using RT-qPCR, it was shown that perilipin2 expression was not altered either in the *gastrocnemius* or in the *soleus*. Moreover, markers of fatty acid oxidation were similar between groups (Sup Figure 2a–h). Finally, we noticed a diet-related downregulation of Cidec mRNA expression in BAT, which was partially counteracted by the treatment. Interestingly, this effect was not observed in the SAT (Figure 4a).

**Figure 5.**
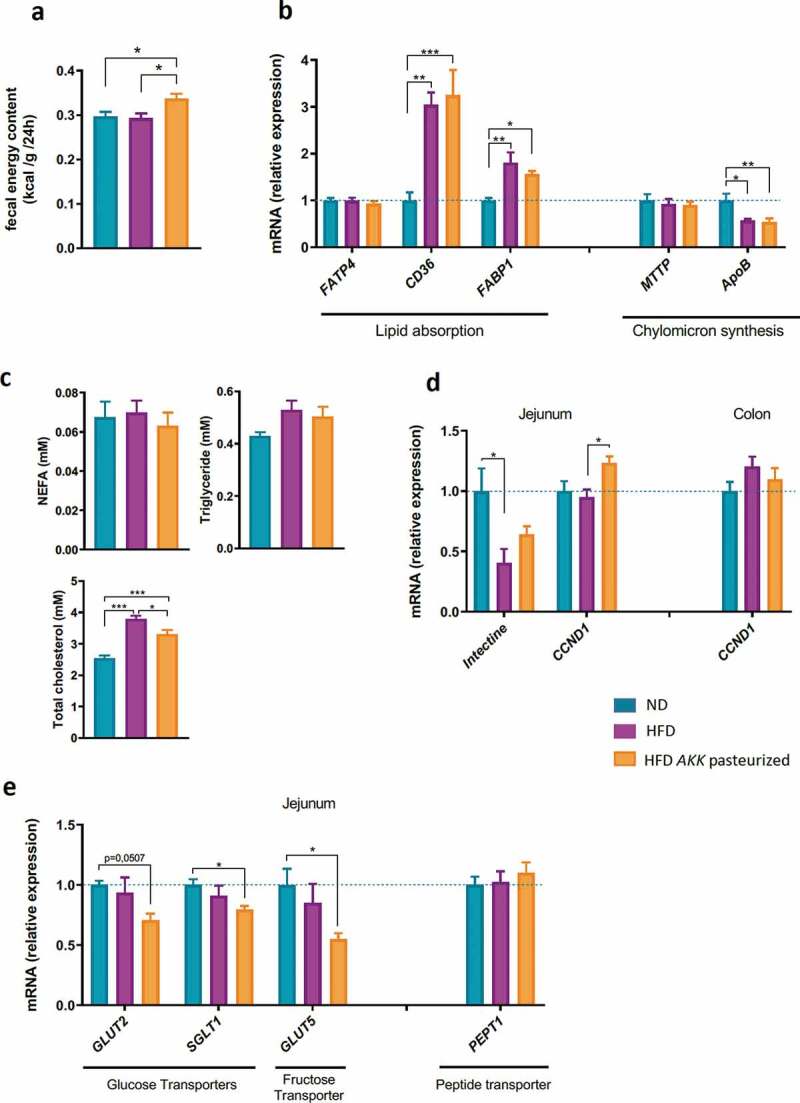
Pasteurized *A. muciniphila* increased fecal energy content without affecting markers of lipid absorption and chylomicron synthesis in the jejunum but modulated carbohydrate absorption and intestinal epithelial cell turnover.

### Pasteurized A. muciniphila increases fecal energy content and reduces expression of carbohydrates transporters

To further explore other physiologic pathways by which energy could have been spent in the treated mice, we measured fecal energy content by calorimetric bomb. We found that pasteurized *A. muciniphila* significantly increased fecal caloric content when compared to both untreated groups (Figure 5a). To decipher the mechanism underlying this effect, markers related to lipid, peptides and carbohydrates absorption were assessed in the jejunum. The results from qPCR analysis demonstrated a significant overexpression of CD36 and FABP1 under HFD conditions. Additionally, while MTTP mRNA expression was not affected by any condition, ApoB mRNA expression was decreased with an HFD (Figure 5b). Interestingly, pasteurized *A. muciniphila* was not able to restore any of those markers to a level comparable to the ND group. Despite alterations in lipid absorption, the treatment successfully blunted HFD-induced hypercholesterolemia. By contrast, plasma triglycerides and nonesterified fatty acids (NEFAs) were unchanged in all groups (Figure 5c). Finally, the transcript levels of the main glucose transporters GLUT2 and SGLT1 mRNA were significantly decreased in the group supplemented with pasteurized *A. muciniphila* when compared to the ND group (Figure 5e). A similar decrease was measured for the fructose transporter GLUT5 mRNA, whereas the expression of PEPT1 mRNA remained unchanged (Figure 5e).

### Pasteurized A. muciniphila modulates markers of intestinal epithelial cell turnover

We next tested if part of the excreted energy could come from enhanced epithelial cell turnover. For this purpose, we quantified the expression of intectin and cyclin D1 (CCDN1), two markers of intestinal cell turn over. Our data showed that intectin expression was significantly reduced by an HFD in jejunum (Figure 5d). Interestingly, this downregulation was partially corrected upon treatment with the pasteurized *A. muciniphila* cells to levels statistically similar to ND mice (Figure 5d). In line with this, pasteurized *A. muciniphila* significantly increased the transcript level of cyclin D1 in the group supplemented with when compared to the HFD-fed group, but this effect was restricted to the jejunum (Figure 5d).

## Discussion

In this study, we confirmed our original finding that pasteurized *A. muciniphila* treatment attenuates DIO in terms of body weight gain and body composition.^23,24^ By using metabolic chambers and indirect calorimetry, we found that the phenotype could be explained by several mechanisms including an enhanced energy expenditure and physical activity and an increased fecal energy excretion in the pasteurized *A. muciniphila* treated mice. Of note, the drop in RER observed with an HFD, which is highlighting an increased oxidation of fat, was not affected by the treatment. However, the observed increase in CO_2_ production and O_2_ consumption clearly corroborates an overall increase in oxidation of fat compared to the HFD groups. When compared to the ND group, locomotor activity was also increased by pasteurized *A. muciniphila*. However, the fact that decreased energy expenditure was observed with an HFD, despite a similar locomotor activity, further supports that divergence in body weight gain could not be entirely attributable to disparities in physical activity. Moreover, we may not rule out that the observed changes in physical activity may simply be secondary to the lower body weight gain observed in pasteurized *A. muciniphila* treated mice.

Accordingly, we next examined markers of two well-known energy producing processes, known to be associated with protection against obesity-related metabolic diseases, called thermogenesis and beiging.^29-31^ We noticed that restoration of the impaired BAT whitening and activity were not the major mechanisms for the energy expenditure-enhancing action of pasteurized *A. muciniphila*. Moreover, the hypothesis of stimulated beiging was excluded, since the defective SAT activity with an HFD was not restored by the treatment. Taken together, these results suggest that the beneficial effect on energy expenditure induced by the pasteurized *A. muciniphila* is independent from BAT and white adipose tissue (WAT) activities. Conversely, recent studies provide strong evidence of positive correlations between the increased abundance of *A. muciniphila* following a bile diversion procedure or supplementation with tea or coffee extract and browning processes.^26,27^ A recent study using a five times higher dose of live *A. muciniphila* than the one used in our study, observed a slight upregulation of UCP-1.^25^ The confrontation of this recent literature with our data acknowledges the previous formulated hypothesis that the live and the pasteurized bacterium might act beneficially but through different metabolic pathways.^24^ Among the various regulators of adipocyte metabolism, lipid droplet-associated proteins have recently appeared to interfere with energy expenditure and obesity-related parameters.^32-34^ Mice overexpressing perilipin1 are obese-resistant and share attractive similarities with our model, notably in terms of enhanced energy expenditure and oxygen consumption.^35,36^ Moreover, obesity is characterized by a reduced expression of perilipin1 in WAT.^37-39^ Importantly, perilipin1 phosphorylation results in increased access of lipase to lipid droplets and stimulated lipolysis.^28,40^ Here, in addition to the lack of HFD effect on its expression, we found that the treatment did not modulate perilipin1, either in terms of mRNA expression or phosphorylated protein. The unaffected *HSL* and *ATGL* expression in SAT corroborates these findings. Nevertheless, we noticed a differential effect of the treatment on the perilipins. While perilipin1 was unaffected by any condition, we observed an adipose-specific perilipin2 downregulation, in parallel with the restoration of Cidec expression in BAT upon pasteurized *A. muciniphila* treatment. Although those data warrants further confirmation at the protein level, it is worth noting that perilipin2 is usually found upregulated in obesity.^41,42^ More strikingly, multiple studies demonstrated that perilipin2 knockout mice developed a highly reproducible obesity-resistant phenotype^43-47^ associated with modulation of the gut microbiota composition.^48^ Finally, Cidec expression was reduced in obese individuals and upregulated following bariatric surgery-induced weight loss.^49,50^

In line with our previous work, we demonstrated that administration of pasteurized *A. muciniphila* cells increased fecal energy content, along with reduced plasma total cholesterol.^23^ To determine the possible mechanism for the current observation, we next examined markers of nutrient absorption. We found that markers of lipid absorption were not affected by the treatment. Along with these findings, we further speculated that lipid utilization and oxidation might be enhanced in peripheral tissues (i.e., muscles). This assumption was excluded, since we did not find any significant differences for genes related to fatty acid oxidation in muscles between the groups. However, we cannot draw definitive conclusions regarding the nonbeneficial effect of the bacterium on fatty acid oxidation or lipolysis, since our model was not characterized by major alterations in these markers. This outcome may be due to the relatively short-term exposure to an HFD (i.e., 5 weeks versus 8–16 weeks).

Importantly, we discovered that pasteurized *A. muciniphila* significantly altered the expression of several major intestinal carbohydrate transporters. Indeed, we found that GLUT2, GLUT5 and SGLT1 mRNA were significantly decreased, thereby suggesting that pasteurized *A. muciniphila* decreases glucose and fructose absorption in the jejunum. Altogether, those results strongly suggest that the increased energy loss observed in the feces might be associated with a reduced absorption of carbohydrates. We can further speculate that those mechanisms might partially account for the improved glucose tolerance observed in previous study upon supplementation of pasteurized *A. muciniphila*.^23^

We have previously shown that an HFD or a western diet reduces the expression of intectin,^51-53^ a marker of intestinal epithelial cell renewal.^54^ In the present study, we found that the increased energy excretion was accompanied by the restoration of the expression of intectin in the jejunum and increased transcript level of Cyclin D1, another marker of intestinal epithelial turnover. Interestingly, we previously discovered that the expression of intectin was enhanced following supplementation with oligofructose,^51,53^ a prebiotic known to increase the abundance of *A. muciniphila* in rodents.^51,55^ Of note, expression of Cyclin D1, among others, is known to be decreased in absence of gut microbiota, supporting the notion that the gut microbiota is able to affect intestinal cell renewal.^56,57^ Although further research is needed, this interesting finding might suggest that pasteurized *A. muciniphila* promotes the turnover of intestinal mucosa throughout the intestinal tract, thereby not only contributing to the maintenance of the gut barrier but also contributing to higher energy loss in the feces via the shedding of intestinal epithelial cells.

In conclusion, by using gold-standard and state-of-the-art methods, we discovered that pasteurized *A. muciniphila* administration reduced body-weight and fat mass gain through several complementary mechanisms such as an increased whole-body energy expenditure, as well as an increased excretion of energy in the feces which can be potentially explained by a higher intestinal epithelial cell turnover as well as a reduction of the absorption of different carbohydrates. Altogether, our data highlight that the protective effect of pasteurized *A. muciniphila* on HFD-induced obesity implies enhanced energy expenditure and physical activity. Moreover, by studying the potential pathways explaining the leakage of energy, we bring to light an interesting effect of the bacteria on carbohydrates absorption, intestinal turnover and possibly on an increased fecal energy content in the treated mice.

## Materials and methods

### Mice and experimental design

C57BL/6J 8-week old male mice (n = 7 per group) were purchased from Janvier (France) and were housed in a specific and opportunistic pathogen free (SOPF) animal facility with a controlled temperature-humidity and 12 h light-dark cycle. Mice had free access to water and food. Upon delivery, mice underwent an acclimatization period of one week during which they were fed with a normal diet (ND) (AIN96Mi, Research diet, New Brunswick, NJ, USA). Mice were matched according to fat mass and body weight and divided into three groups. During the following 5 weeks, mice were fed a ND or an HFD (60% fat and 20% carbohydrates (kcal per 100 g), D12492i, Research diet New Brunswick, NJ, USA) and were treated daily with an oral gavage of either 2 × 10^8^ CFU/180 µl of pasteurized *A. muciniphila* (ATTC BAA-835) in sterile PBS containing 2.5% glycerol or 180 µl of vehicle solution (PBS containing 2.5% glycerol). Pasteurization consisted of heat treatment at 70°C for 30 min of fresh *A. muciniphila* produced, as previously described.^23^ Body weight and body composition were measured once a week using 7.5 mHz TD-NMR (LF50 minispec, Bruker). Food intake was assessed weekly. Cumulative food intake and energy efficiency were calculated based on the 4 first weeks. Mice were separated one week before indirect calorimetry measurement for acclimation. In the final week of the experiment, fecal samples were harvested after a 48 h period and fecal energy content was measured using a bomb calorimeter (Staufen, Germany). All data shown are compiled from two independent experiment (*n = 3–4* for each set of experiment, leading to n = 7/group in total).

All mouse experiments were approved by and performed in accordance with the guideline of the local ethics committee (Ethics Committee of the Université Catholique de Louvain for Animal Experiments specifically approved this study that received the agreement number 2017/UCL/MD/005). Housing conditions were specified by the Belgian Law of 29 May 2013, regarding the protection of laboratory animals (agreement number LA1230314).

### Indirect calorimetry study

At 4 weeks of treatment, the mice were placed individually in metabolic chambers (Labmaster, TSE systems GmbH, Bad Homburg, Germany) for seven days. Mice were housed with free access to food and water (ND or HFD) and were acclimated to the chambers for 24 h before experimental measurements. The mice were analyzed for RER (calculated as vCO2/vO2), whole energy expenditure, oxygen consumption, and carbon dioxide production using indirect calorimetry (Labmaster, TSE systems GmbH). The last three parameters were expressed as a function of whole-body weight. Locomotor activity was recorded using an infrared light beam-based locomotion monitoring system (expressed as Beam Breaks count per hour). The measurement period accounted for six and a half days in total, starting at 6 pm the first day and finishing at 6 am the last day. Inside the chambers, measurements were taken every 15 minutes. Values were then summed by hour. For each mouse, a mean value was obtained for every hour of a 24 h day cycle and was calculated from the basis of 6 different measurements (6 days of measurements). Accordingly, the final data representation (total, day or night) corresponds to all the values measured and summed (light phase or dark phase). The means (*n = 7*) were finally compared between groups.

### Tissue sampling

At the end of the treatment period, the animals were anesthetized with isoflurane (Forene; Abbott). Blood was sampled from the cava vein. After exsanguination and tissue sampling, the mice were killed by decapitation. Adipose depots (subcutaneous, epididymal, mesenteric and brown) were precisely dissected and weighed. The intestinal segments, muscles (*gastrocnemius* and *soleus*) and adipose tissues were immersed in liquid nitrogen and stored at −80°C for further analysis. Pieces of BAT were sampled for further histological analyses.

### Histological analysis

BAT depots were fixed in 4% paraformaldehyde for 24 h at room temperature before processing for paraffin embedding. Hematoxylin and eosin staining was performed using standard protocols on 5-μm tissue sections. Images were obtained using a SCN400 slide scanner and Digital Image Hub software (Leica Biosystems, Wetzlar, Germany) and were captured using Leica Image Viewer Software (National Institutes of Health, Bethesda, Maryland, USA). A minimum of 5 high-magnification fields were analyzed per mouse.

### RNA preparation and real-time qPCR analysis

Total RNA was prepared from collected tissues by using TriPure reagent from Roche. cDNA was prepared by reverse transcription of 1 µg total RNA using a Reverse Transcription System Kit (Promega, Madison, Wisconsin, USA). Real-time PCR was performed with the CFX96 Real-time PCR system and CFX manager 3.1 software (Bio-Rad, Hercules, California, USA) using GoTaq qPCR Master Mix (Promega) for detection, according to the manufacturer’s instructions. RPL19 RNA was chosen as the housekeeping gene, and data were analyzed according to the 2^−ΔΔCT^ method. The identity and purity of the amplified product were assessed by melting curve analysis at the end of amplification. The primer sequences for the targeted mouse genes are presented in Table 1.

**Table 1.** qPCR primer sequences for the targeted mouse genes.

Primers	Forward Sequence	Reverse Sequence
UCP-1	GCTACACGGGGACCTACAATG	CGTCATCTGCCAGTATTTTGTT
Elovl6	AAAGCACCCGAACTAGGTGA	AGGAGCACAGTGATGTGGTG
Elovl3	TTCTCACGCGGGTTAAAAATGG	GAGCAACAGATAGACGACCAC
Cidea	GCAGCCTGCAGGAACTTATC	TCATGAAATGCGTGTTGTCC
Dio2	AATTATGCCTCGGAGAAGACCG	GGCAGTTGCCTAGTGAAAGGT
PGC1a	AGCCGTGACCACTGACAACGAG	GCTGCATGGTTCTGAGTGCTAAG
ACO	CTATGGGATCAGCCAGAAAGG	AGTCAAAGGCATCCACCAAAG
TBX1	TCGACAAGCTGAAACTGACC	GCGTGTCTCCTCAAACACAA
CD137	ATGCTTGTAGCTGCCGATGT	GGTGGGGTCCTAGTGCTTCT
HSL	GCTAGCCAGGCTCATCTCCT	GTTCTTGAGGTAGGGCTCGT
ATGL	ACAGCTCCAACATCCAC	AGCCCTGTTTGCACATCTCT
Cidec	GCACAATCGTGGAGACAGAA	TAGTTGGCTTCTGGGAAAGG
Plin1	GATGCCCTGAAGGGTGTTAC	CCTCTGCTGAAGGGTTATCG
Plin2	CTCCACTCCACTGTCCACCT	CGATGCTTCTCTTCCACTCC
FATP4	TTGCAAGTCCCATCAGCAAC	AACAGCGGCTCTTTCACAAC
CD36	ATCTCAATGTCCGAGACTTTTCAAC	GCCAAGCTATTGGGACATGA
FABP1	TGATGTCCTTCCCTTTCTGG	GCAGAGCCAGGAGAACTTTG
Intectin	GTTGCCCCTGATTCTGCTGG	GCACTATTGCAGAGGTCCGT
CCND1	CGGATGAGAACAAGCAGACC	AGGGTGGGTTGGAAATGAAC
GLUT2	AGAGATCGCTCCAACCACAC	AATGATCCTGATTGCCCAGA
GLUT5	AGCCATCAACAAGGCAGAAC	CTGCCGTAGAAGACACCACA
SGLT1	TCTTCGTCATCAGCGTCATC	AGGTCGATTCGCTCTTCCTT
PEPT1	CGCTTGCCCAAATGTCTC	CGGTGACCCTGCTCAAAA
RPL19	GAAGGTCAAAGGGAATGTGTTCA	CCTTGTCTGCCTTCAGCTTGT

### Western blotting

Subcutaneous adipose tissues were homogenized in RIPA buffer (Tris HCL 25 mM, NaCl 150 mM, 1% NP-40, 0,1% sodium dodecyl sulfate) supplemented with a cocktail of protease inhibitors and phosphatase inhibitors. Equal amounts of proteins were separated by SDS–PAGE and transferred to nitrocellulose membranes. Membranes were incubated 1 h at room temperature with anti-phospho-perilipin1 (1:5000, Vala Science, #4856) and overnight at 4°C with anti-perilipin1 (1:1000, Cell signaling #9349). Both antibodies were diluted in Tris-buffered saline-Tween-20 containing 1% bovine serum albumin. The loading control was HSP90 (1:500, sc-13119, Santa Cruz). Signal quantification was acquired with an Amersham Imager 600 (GE Healthcare) and analyzed by ImageQuant TL software.

### Biochemical analysis

Plasma triglycerides and plasma total cholesterol were measured in cava blood using kits coupling the enzymatic reaction and spectrophotometric detection of the final product (Diasys Diagnostic and System, Holzheim, Germany). Plasma non-esterified fatty acid levels were assessed using a commercially available enzymatic assay (Randox Laboratories, Crumlin, UK).

### Statistical analysis

The data are presented as the means ± s.e.m. The statistical significance of difference was evaluated by one-way or two-way ANOVA followed by Tukey’s *post hoc* multiple comparison test. Analysis of covariance (ANCOVA) with the lean mass (g) as covariable, was performed on data relative to energy expenditure, VO_2_ and CO_2_ consumption divided by body weight. For ANCOVA, assumptions of homogeneity of regression and independence of the covariate to the treatment effects were respected. The data with a superscript symbol (* vs ND, # vs HFD) are significantly different (*p < .05; **p < .01; ***p < .001). Except ANCOVA, all the analyses were performed using GraphPad Prism version 8.00 for Windows (GraphPad Software). ANCOVA was conducted using SPSS v.23.0 (IBM Corporation). The presence of outliers was assessed using the Grubbs test.

## Supplementary Material

Supplemental Material

## References

[1] Collaboration NCDRF. Trends in adult body-mass index in 200 countries from 1975 to 2014: a pooled analysis of 1698 population-based measurement studies with 19.2 million participants. Lancet. 2016;387:1377–1396. doi:10.1016/S0140-6736(16)30054-X. PMID: 27115820. 27115820 PMC7615134

[2] O’Neill S, O’Driscoll L. Metabolic syndrome: a closer look at the growing epidemic and its associated pathologies. Obes Rev 2015;16:1–12. doi:10.1111/obr.12229. PMID: 25407540. 25407540

[3] Cani PD, Van Hul M, Lefort C, Depommier C, Rastelli M, Everard A. Microbial regulation of organismal energy homeostasis. Nature Metab 2019;1:34–46. doi:10.1038/s42255-018-0017-4. PMID. 32694818

[4] Rastelli M, Knauf C, Cani PD. Gut microbes and health: a focus on the mechanisms linking microbes, obesity, and related disorders. Obesity (Silver Spring) 2018;26:792–800. doi:10.1002/oby.22175. PMID: 29687645. 29687645 PMC5947576

[5] Rosenbaum M, Knight R, Leibel RL. The gut microbiota in human energy homeostasis and obesity. Trends Endocrinol Metab 2015;26:493–501. doi:10.1016/j.tem.2015.07.002. PMID: 26257300. 26257300 PMC4862197

[6] Khan MT, Nieuwdorp M, Backhed F. Microbial modulation of insulin sensitivity. Cell Metab 2014;20:753–760. doi:10.1016/j.cmet.2014.07.006. PMID: 25176147. 25176147

[7] Cani PD, de Vos WM. Next-generation beneficial microbes: the case of Akkermansia muciniphila. Front Microbiol 2017;8:1765. doi:10.3389/fmicb.2017.01765. PMID: 29018410. 29018410 PMC5614963

[8] Derrien M, Belzer C, de Vos WM. Akkermansia muciniphila and its role in regulating host functions. Microb Pathog 2017;106:171–181. doi:10.1016/j.micpath.2016.02.005. PMID: 26875998. 26875998

[9] Dao MC, Everard A, Aron-Wisnewsky J, Sokolovska N, Prifti E, Verger EO, Kayser BD, Levenez F, Chilloux J, Hoyles L, et al. Akkermansia muciniphila and improved metabolic health during a dietary intervention in obesity: relationship with gut microbiome richness and ecology. Gut 2016;65:426–436. doi:10.1136/gutjnl-2014-308778. PMID: 26100928. 26100928

[10] Brahe LK, Le Chatelier E, Prifti E, Pons N, Kennedy S, Hansen T, Pedersen O, Astrup A, Ehrlich SD, Larsen LH, et al. Specific gut microbiota features and metabolic markers in postmenopausal women with obesity. Nutr Diabetes 2015;5:e159. doi:10.1038/nutd.2015.9. PMID: 26075636. 26075636 PMC4491860

[11] Le Chatelier E, Nielsen T, Qin J, Prifti E, Hildebrand F, Falony G, Almeida M, Arumugam M, Batto J-M, Kennedy S, et al. Richness of human gut microbiome correlates with metabolic markers. Nature 2013;500:541–546. doi:10.1038/nature12506. PMID: 23985870. 23985870

[12] Zhang X, Shen D, Fang Z, Jie Z, Qiu X, Zhang C, Chen Y, Ji L. Human gut microbiota changes reveal the progression of glucose intolerance. PLoS One 2013;8:e71108. doi:10.1371/journal.pone.0071108. PMID: 24013136. 24013136 PMC3754967

[13] Yassour M, Lim MY, Yun HS, Tickle TL, Sung J, Song Y-M, Lee K, Franzosa EA, Morgan XC, Gevers D. Sub-clinical detection of gut microbial biomarkers of obesity and type 2 diabetes. Genome Med 2016;8:17. doi:10.1186/s13073-016-0271-6. PMID: 26884067. 26884067 PMC4756455

[14] Png CW, Linden SK, Gilshenan KS, Zoetendal EG, McSweeney CS, Sly LI, McGuckin MA, Florin THJ. Mucolytic bacteria with increased prevalence in IBD mucosa augment in vitro utilization of mucin by other bacteria. American Journal of Gastroenterology 2010;105:2420–2428. doi:10.1038/ajg.2010.281. PMID: 20648002. 20648002

[15] Peeters T, Penders J, Smeekens SP, Galazzo G, Houben B, Netea MG, Savelkoul PH, Gyssens IC. The fecal and mucosal microbiome in acute appendicitis patients: an observational study. Future Microbiology 2019;14:111–127. doi:10.2217/fmb-2018-0203. PMID: 30663346. 30663346

[16] Everard A, Belzer C, Geurts L, Ouwerkerk JP, Druart C, Bindels LB, Guiot Y, Derrien M, Muccioli GG, Delzenne NM. Cross-talk between Akkermansia muciniphila and intestinal epithelium controls diet-induced obesity. Proc Natl Acad Sci U S A 2013;110:9066–9071. doi:10.1073/pnas.1219451110. PMID: 23671105. 23671105 PMC3670398

[17] Li J, Lin S, Vanhoutte PM, Woo CW, Xu A. Akkermansia muciniphila protects against atherosclerosis by preventing metabolic endotoxemia-induced inflammation in Apoe-/- Mice. Circulation 2016;133:2434–2446. doi:10.1161/CIRCULATIONAHA.115.019645. PMID: 27143680. 27143680

[18] Wu W, Lv L, Shi D, Ye J, Fang D, Guo F, Li Y, He X, Li L. Protective effect of Akkermansia muciniphila against immune-mediated liver injury in a mouse model. Front Microbiol 2017;8:1804. doi:10.3389/fmicb.2017.01804. PMID: 29033903. 29033903 PMC5626943

[19] Shin NR, Lee JC, Lee HY, Kim MS, Whon TW, Lee MS, Bae J-W. An increase in the Akkermansia spp. population induced by metformin treatment improves glucose homeostasis in diet-induced obese mice. Gut 2014;63:727–735. doi:10.1136/gutjnl-2012-303839. PMID: 23804561. 23804561

[20] Hanninen A, Toivonen R, Poysti S, Belzer C, Plovier H, Ouwerkerk JP. Akkermansia muciniphila induces gut microbiota remodelling and controls islet autoimmunity in NOD mice. Gut. 2017. doi:10.1136/gutjnl-2017-314508. PMID: 29269438. 29269438

[21] van der Lugt B, van Beek AA, Aalvink S, Meijer B, Sovran B, Vermeij WP, Brandt RMC, de Vos WM, Savelkoul HFJ, Steegenga WT. Akkermansia muciniphila ameliorates the age-related decline in colonic mucus thickness and attenuates immune activation in accelerated aging Ercc1 (-/Delta7) mice. Immun Ageing 2019;16:6. doi:10.1186/s12979-019-0145-z. PMID: 30899315. 30899315 PMC6408808

[22] Grander C, Adolph TE, Wieser V, Lowe P, Wrzosek L, Gyongyosi B. Recovery of ethanol-induced Akkermansia muciniphila depletion ameliorates alcoholic liver disease. Gut 2018;67:891–901. doi:10.1136/gutjnl-2016-313432. PMID: 28550049. 10.1136/gutjnl-2016-313432 28550049

[23] Plovier H, Everard A, Druart C, Depommier C, Van Hul M, Geurts L, Chilloux J, Ottman N, Duparc T, Lichtenstein L, et al. A purified membrane protein from Akkermansia muciniphila or the pasteurized bacterium improves metabolism in obese and diabetic mice. Nat Med 2017;23:107–113. doi:10.1038/nm.4236. PMID: 27892954. 27892954

[24] Depommier C, Everard A, Druart C, Plovier H, Van Hul M, Vieira-Silva S, Falony G, Raes J, Maiter D, Delzenne NM, et al. Supplementation with Akkermansia muciniphila in overweight and obese human volunteers: a proof-of-concept exploratory study. Nat Med 2019;25:1096–1103. doi:10.1038/s41591-019-0495-2. PMID: 31263284. 31263284 PMC6699990

[25] Gao X, Xie Q, Kong P, Liu L, Sun S, Xiong B. Polyphenol- and caffeine-rich postfermented Pu-erh tea improves diet-induced metabolic syndrome by remodeling intestinal homeostasis in mice. Infect Immun. 2018;86. doi:10.1128/IAI.00601-17.PMID: 29061705. PMC5736808 29061705

[26] Liu J, Li Y, Yang P, Wan J, Chang Q, Wang TTY. Gypenosides reduced the risk of overweight and insulin resistance in C57BL/6J mice through modulating adipose thermogenesis and gut microbiota. J Agric Food Chem. 2017. doi:10.1021/acs.jafc.7b03382. PMID: 28975783. 28975783

[27] Pierre JF, Martinez KB, Ye H, Nadimpalli A, Morton TC, Yang J, Wang Q, Patno N, Chang EB, Yin DP, et al. Activation of bile acid signaling improves metabolic phenotypes in high-fat diet-induced obese mice. Am J Physiol Gastrointest Liver Physiol 2016;311:G286–304. doi:10.1152/ajpgi.00202.2016. PMID: 27340128. 27340128 PMC5007288

[28] Sztalryd C, Brasaemle DL. The perilipin family of lipid droplet proteins: gatekeepers of intracellular lipolysis. Biochim Biophys Acta Mol Cell Biol Lipids 2017;1862:1221–1232. doi:10.1016/j.bbalip.2017.07.009. PMID: 28754637. 28754637 PMC5595658

[29] Li H, Zhang C, Liu J, Xie W, Xu W, Liang F, Huang K, He X. Intraperitoneal administration of follistatin promotes adipocyte browning in high-fat diet-induced obese mice. PLoS One 2019;14:e0220310. doi:10.1371/journal.pone.0220310. PMID: 31365569. 31365569 PMC6668797

[30] Neyrinck AM, Bindels LB, Geurts L, Van Hul M, Cani PD, Delzenne NM. A polyphenolic extract from green tea leaves activates fat browning in high-fat-diet-induced obese mice. J Nutr Biochem 2017;49:15–21. doi:10.1016/j.jnutbio.2017.07.008. PMID: 28863365. 28863365

[31] Han X, Guo J, You Y, Yin M, Liang J, Ren C, Zhan J, Huang W. Vanillic acid activates thermogenesis in brown and white adipose tissue. Food Funct 2018;9:4366–4375. doi:10.1039/c8fo00978c. PMID: 30043820. 30043820

[32] Bickel PE, Tansey JT, Welte MA. PAT proteins, an ancient family of lipid droplet proteins that regulate cellular lipid stores. Biochim Biophys Acta 2009;1791:419–440. doi:10.1016/j.bbalip.2009.04.002. PMID: 19375517. 19375517 PMC2782626

[33] Sohn JH, Lee YK, Han JS, Jeon YG, Kim JI, Choe SS, Kim SJ, Yoo HJ, Kim JB. Perilipin 1 (Plin1) deficiency promotes inflammatory responses in lean adipose tissue through lipid dysregulation. J Biol Chem 2018;293:13974–13988. doi:10.1074/jbc.RA118.003541. PMID: 30042231. 30042231 PMC6130955

[34] Traini M, Jessup W. Lipid droplets and adipose metabolism: a novel role for FSP27/CIDEC. Curr Opin Lipidol 2009;20:147–149. doi:10.1097/MOL.0b013e32832956c7. PMID: 19276894. 19276894

[35] Miyoshi H, Souza SC, Endo M, Sawada T, Perfield JW 2nd, Shimizu C, Stancheva Z, Nagai S, Strissel KJ, Yoshioka N. Perilipin overexpression in mice protects against diet-induced obesity. J Lipid Res 2010;51:975–982. doi:10.1194/jlr.M002352. PMID: 19797618. 19797618 PMC2853465

[36] Sawada T, Miyoshi H, Shimada K, Suzuki A, Okamatsu-Ogura Y, Perfield JW 2nd, Kondo T, Nagai S, Shimizu C, Yoshioka N, et al. Perilipin overexpression in white adipose tissue induces a brown fat-like phenotype. PLoS One 2010;5:e14006. doi:10.1371/journal.pone.0014006. PMID: 21103377. 21103377 PMC2982838

[37] Ju L, Han J, Zhang X, Deng Y, Yan H, Wang C, Li X, Chen S, Alimujiang M, Li X, et al. Obesity-associated inflammation triggers an autophagy-lysosomal response in adipocytes and causes degradation of perilipin 1. Cell Death Dis 2019;10:121. doi:10.1038/s41419-019-1393-8. PMID: 30741926. 30741926 PMC6370809

[38] Gaidhu MP, Anthony NM, Patel P, Hawke TJ, Ceddia RB. Dysregulation of lipolysis and lipid metabolism in visceral and subcutaneous adipocytes by high-fat diet: role of ATGL, HSL, and AMPK. Am J Physiol Cell Physiol 2010;298:C961–71. doi:10.1152/ajpcell.00547.2009. PMID: 20107043. 20107043

[39] Wang Y, Sullivan S, Trujillo M, Lee MJ, Schneider SH, Brolin RE, Kang YH, Werber Y, Greenberg AS, Fried SK, et al. Perilipin expression in human adipose tissues: effects of severe obesity, gender, and depot. Obes Res 2003;11:930–936. doi:10.1038/oby.2003.128. PMID: 12917496. 12917496

[40] McDonough PM, Maciejewski-Lenoir D, Hartig SM, Hanna RA, Whittaker R, Heisel A, Nicoll JB, Buehrer BM, Christensen K, Mancini MG, et al. Differential phosphorylation of perilipin 1A at the initiation of lipolysis revealed by novel monoclonal antibodies and high content analysis. PLoS One 2013;8:e55511. doi:10.1371/journal.pone.0055511. PMID: 23405163. 23405163 PMC3566132

[41] Liu LF, Shen WJ, Ueno M, Patel S, Azhar S, Kraemer FB. Age-related modulation of the effects of obesity on gene expression profiles of mouse bone marrow and epididymal adipocytes. PLoS One 2013;8:e72367. doi:10.1371/journal.pone.0072367. PMID: 23967297. 23967297 PMC3743818

[42] Coats BR, Schoenfelt KQ, Barbosa-Lorenzi VC, Peris E, Cui C, Hoffman A, Zhou G, Fernandez S, Zhai L, Hall BA, et al. Metabolically activated adipose tissue macrophages perform detrimental and beneficial functions during diet-induced obesity. Cell Rep 2017;20:3149–3161. doi:10.1016/j.celrep.2017.08.096. PMID: 28954231. 28954231 PMC5646237

[43] Imai Y, Varela GM, Jackson MB, Graham MJ, Crooke RM, Ahima RS. Reduction of hepatosteatosis and lipid levels by an adipose differentiation-related protein antisense oligonucleotide. Gastroenterology 2007;132:1947–1954. doi:10.1053/j.gastro.2007.02.046. PMID: 17484887. 17484887

[44] Varela GM, Antwi DA, Dhir R, Yin X, Singhal NS, Graham MJ, Crooke RM, Ahima RS. Inhibition of ADRP prevents diet-induced insulin resistance. Am J Physiol Gastrointest Liver Physiol 2008;295:G621–8. doi:10.1152/ajpgi.90204.2008. PMID: 18669627. 18669627 PMC2536783

[45] McManaman JL, Bales ES, Orlicky DJ, Jackman M, MacLean PS, Cain S, Crunk AE, Mansur A, Graham CE, Bowman TA. Perilipin-2-null mice are protected against diet-induced obesity, adipose inflammation, and fatty liver disease. J Lipid Res 2013;54:1346–1359. doi:10.1194/jlr.M035063. PMID: 23402988. 23402988 PMC3622329

[46] Najt CP, Senthivinayagam S, Aljazi MB, Fader KA, Olenic SD, Brock JR, Lydic TA, Jones AD, Atshaves BP. Liver-specific loss of Perilipin 2 alleviates diet-induced hepatic steatosis, inflammation, and fibrosis. Am J Physiol Gastrointest Liver Physiol 2016;310:G726–38. doi:10.1152/ajpgi.00436.2015. PMID: 26968211. 26968211 PMC4867327

[47] Libby AE, Bales ES, Monks J, Orlicky DJ, McManaman JL. Perilipin-2 deletion promotes carbohydrate-mediated browning of white adipose tissue at ambient temperature. J Lipid Res 2018;59:1482–1500. doi:10.1194/jlr.M086249. PMID: 29866659. 29866659 PMC6071773

[48] Frank DN, Bales ES, Monks J, Jackman MJ, MacLean PS, Ir D, Robertson CE, Orlicky DJ, McManaman JL. Perilipin-2 modulates lipid absorption and microbiome responses in the mouse intestine. PLoS One 2015;10:e0131944. doi:10.1371/journal.pone.0131944. PMID: 26147095. 26147095 PMC4493139

[49] Moreno-Navarrete JM, Ortega F, Serrano M, Rodriguez-Hermosa JI, Ricart W, Mingrone G. CIDEC/FSP27 and PLIN1 gene expression run in parallel to mitochondrial genes in human adipose tissue, both increasing after weight loss. Int J Obes (Lond) 2014;38:865–872. doi:10.1038/ijo.2013.171. PMID: 24126816. 24126816

[50] Keller P, Petrie JT, De Rose P, Gerin I, Wright WS, Chiang S-H, Nielsen AR, Fischer CP, Pedersen BK, MacDougald OA, et al. Fat-specific protein 27 regulates storage of triacylglycerol. J Biol Chem 2008;283:14355–14365. doi:10.1074/jbc.M708323200. PMID: 18334488. 18334488 PMC2386939

[51] Everard A, Lazarevic V, Gaia N, Johansson M, Stahlman M, Backhed F, Delzenne NM, Schrenzel J, François P, Cani PD, et al. Microbiome of prebiotic-treated mice reveals novel targets involved in host response during obesity. Isme J 2014;8:2116–2130. doi:10.1038/ismej.2014.45. PMID: 24694712. 24694712 PMC4163056

[52] Van Hul M, Geurts L, Plovier H, Druart C, Everard A, Stahlman M, et al. Reduced obesity, diabetes and steatosis upon cinnamon and grape pomace are associated with changes in gut microbiota and markers of gut barrier. Am J Physiol Endocrinol Metab 2017; ajpendo 00107 2017. doi:10.1152/ajpendo.00107.2017. PMID: 28874357. 28874357

[53] Olivares M, Rodriguez J, Potgens SA, Neyrinck AM, Cani PD, Bindels LB, Delzenne NM. The janus face of cereals: wheat-derived prebiotics counteract the detrimental effect of gluten on metabolic homeostasis in mice fed a high-fat/high-sucrose diet. Mol Nutr Food Res 2019;63:e1900632. doi:10.1002/mnfr.201900632. PMID: 31608562. 31608562 PMC7003472

[54] Kitazawa H, Nishihara T, Nambu T, Nishizawa H, Iwaki M, Fukuhara A, Kitamura T, Matsuda M, Shimomura I. Intectin, a novel small intestine-specific glycosylphosphatidylinositol-anchored protein, accelerates apoptosis of intestinal epithelial cells. J Biol Chem 2004;279:42867–42874. doi:10.1074/jbc.M408047200. PMID: 15292182. 15292182

[55] Everard A, Lazarevic V, Derrien M, Girard M, Muccioli GG, Neyrinck AM, Possemiers S, Van Holle A, François P, de Vos WM. Responses of gut microbiota and glucose and lipid metabolism to prebiotics in genetic obese and diet-induced leptin-resistant mice. Diabetes 2011;60:2775–2786. doi:10.2337/db11-0227. PMID. 21933985 PMC3198091

[56] Hormann N, Brandao I, Jackel S, Ens N, Lillich M, Walter U, Reinhardt C. Gut microbial colonization orchestrates TLR2 expression, signaling and epithelial proliferation in the small intestinal mucosa. PLoS One 2014;9:e113080. doi:10.1371/journal.pone.0113080. PMID: 25396415. 25396415 PMC4232598

[57] Park JH, Kotani T, Konno T, Setiawan J, Kitamura Y, Imada S, Usui Y, Hatano N, Shinohara M, Saito Y. Promotion of intestinal epithelial cell turnover by commensal bacteria: role of short-chain fatty acids. PLoS One 2016;11:e0156334. doi:10.1371/journal.pone.0156334. PMID: 27232601. 27232601 PMC4883796

