# Supplementary material for: Pasteurized Akkermansia muciniphila increases whole-body energy expenditure and fecal energy excretion in diet-induced obese mice
Source: Gut Microbes. 2020 Mar 13;11(5):1231–45. doi: 10.1080/19490976.2020.1737307 (PMC7524283; doi:10.1080/19490976.2020.1737307)
Supplement: Supplemental Material [file KGMI_A_1737307_SM4945.zip › Supplementary information/Supplementary figure captions.docx]

**Sup Figure 1.** Impact of diet and pasteurized *A. muciniphila* on lean mass.

**(a-b)** Lean mass evolution expressed in absolute (g) **(a)** and in percentage according to total body weight (%) **(b)** over a 5-week period. Mean + s.e.m, *p<0.05, **p<0.01, ***p<0,001 compared to ND, ^#^p<0.05, ^##^p<0.01, ^###^p<0.001 compared to HFD (*n=7*). Data were analyzed using two-way ANOVA followed by Tukey’s *post hoc* test.

**Sup Figure 2.** HFD and pasteurized *A. muciniphila* does not influence lipid metabolism in muscles.

**(a–d)** mRNA expression of perilipin2 (Plin2), PGC1a, Cpt1a and, ACO in soleus (*n=6–7*). **(e, f)** mRNA expression of Plin2 and PGC1a, **(e, f)** in gastrocnemius (*n=6–7*). Blue: ND mice, pink: HFD mice and orange: HFD mice supplemented with pasteurized *A. muciniphila*. Data are presented as the mean + s.e.m. Data were analyzed by one-way ANOVA followed by Tukey’s *post hoc* test
