# Supplementary figures and images for: Pasteurized Akkermansia muciniphila increases whole-body energy expenditure and fecal energy excretion in diet-induced obese mice
Source: Gut Microbes. 2020 Mar 13;11(5):1231–45. doi: 10.1080/19490976.2020.1737307 (PMC7524283; doi:10.1080/19490976.2020.1737307)

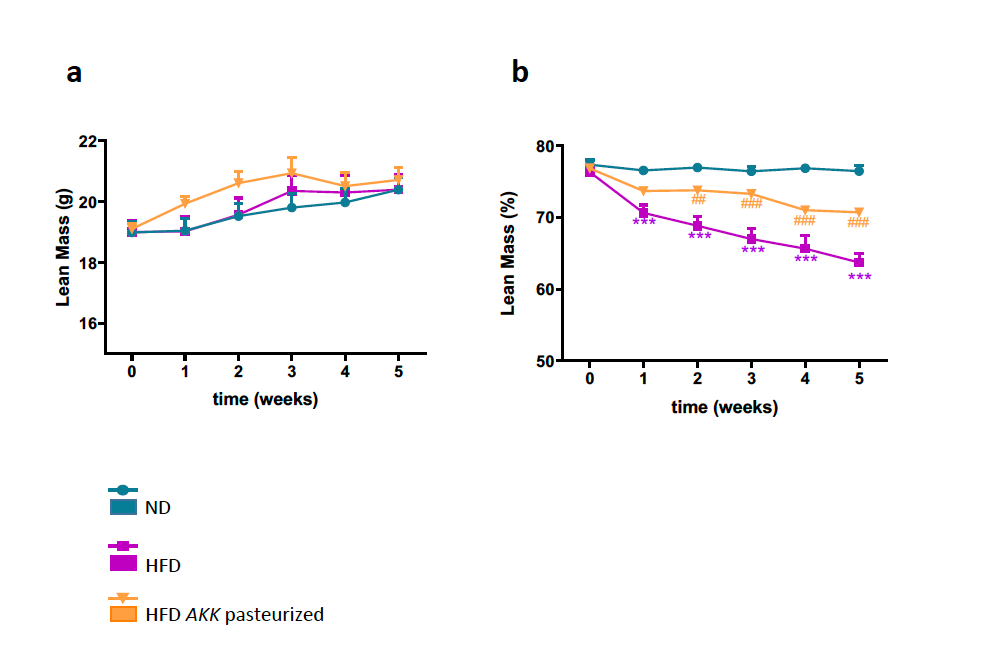

Supplement: Supplemental Material [file KGMI_A_1737307_SM4945.zip › Supplementary information/sup_fig1.tiff]

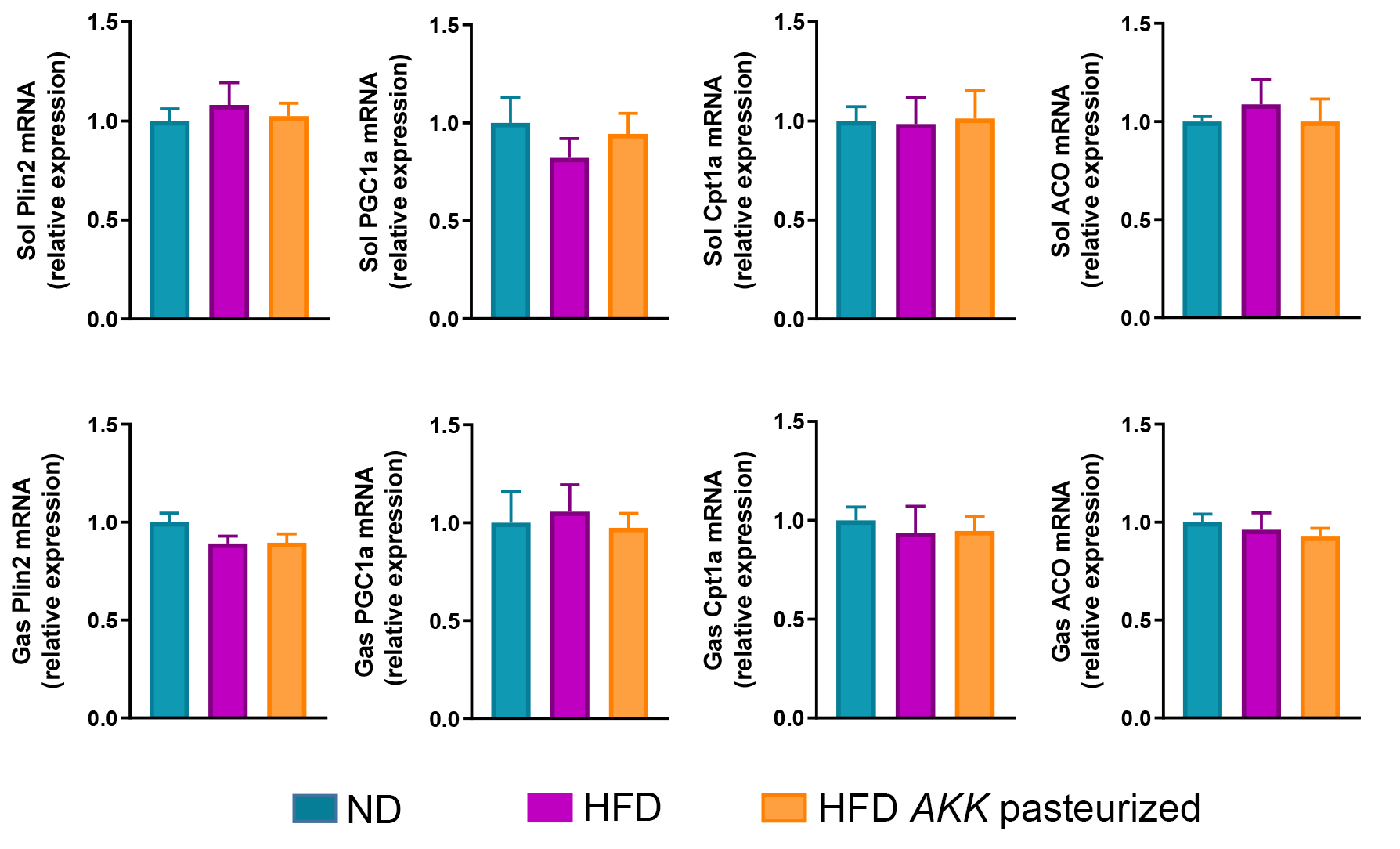

Supplement: Supplemental Material [file KGMI_A_1737307_SM4945.zip › Supplementary information/sup_fig2.tiff]
